# Altered Functional Specialization and Interhemispheric Coordination in Rhegmatogenous Retinal Detachment: Associations With Gene Expression, Neurotransmitter Receptor Distribution, and SVM–SHAP Classification: A Multimodal Neuroimaging–Transcriptomics Study Integrating Functional Metrics and Interpretable Machine Learning

**DOI:** 10.1002/cns.70678

**Published:** 2026-01-07

**Authors:** Yu Ji, Yuan‐Yuan Wang, Xiao‐Rong Wu

**Affiliations:** ^1^ Department of Ophthalmology, The First Affiliated Hospital, Jiangxi Medical College Nanchang University Nanchang Jiangxi China; ^2^ Department of Radiology, The First Affiliated Hospital, Jiangxi Medical College Nanchang University Nanchang Jiangxi China

**Keywords:** Allen Human Brain Atlas, autonomy index, connectivity between functionally homotopic voxels, neurotransmitters receptors, rhegmatogenous retinal detachment, Shapley additive explanations, support vector machine

## Abstract

**Background:**

Previous studies have reported functional alterations in the brains of patients with rhegmatogenous retinal detachment (RRD). However, it remains largely unclear whether RRD affects hemispheric specialization and interhemispheric coordination, and how these alterations relate to underlying gene expression patterns and neurotransmitter receptor distributions.

**Methods:**

We employed the Autonomy Index (AI) and Connectivity between Functionally Homotopic Voxels (CFH) to quantify alterations in hemispheric specialization and interhemispheric cooperation in patients with RRD. Transcriptome–neuroimaging spatial correlation analysis was performed by integrating gene expression data from the Allen Human Brain Atlas (AHBA) to identify genes associated with AI and CFH alterations. Enrichment and protein–protein interaction analyses were conducted to characterize the biological processes and molecular features of these genes. Furthermore, we explored the spatial associations between AI/CFH abnormalities and neurotransmitter receptor distributions. Finally, a support vector machine (SVM) classifier combined with Shapley additive explanations (SHAP) was implemented to distinguish RRD patients from healthy controls (HCs) and to determine the most discriminative brain regions.

**Results:**

RRD patients exhibited significant alterations in AI and CFH within the frontal lobe, occipital lobe, and thalamus. Transcriptome–neuroimaging integration revealed gene sets closely associated with these abnormalities. These genes were primarily enriched in key biological processes including synaptic signaling, sensory organ development, Notch signaling, and structural neuroplasticity. The spatial pattern of CFH changes showed strong alignment with the regional distributions of multiple neurotransmitter systems, particularly serotonergic, dopaminergic, glutamatergic, and cholinergic pathways. Finally, the SVM–SHAP classification framework identified CFH in the right thalamus as the most discriminative feature for differentiating RRD patients from HCs.

**Conclusion:**

These findings deepen our neurobiological understanding of RRD‐induced brain functional remodeling and provide theoretical support and a methodological foundation for developing central intervention strategies and potential discriminative imaging tools for retinal diseases.

## Graphical abstract

Autonomy Index (AI) and Connective Flexibility of Homotopic voxels (CFH) were derived from resting‐state fMRI data following standard preprocessing. These functional metrics were computed based on the AAL90 atlas to characterize intrinsic activity and interhemispheric coordination patterns in RRD patients. Using the Allen Human Brain Atlas (AHBA), regional gene expression profiles from the left hemisphere were aligned to the AAL90 template, generating a gene expression matrix encompassing 15,632 genes for transcriptomic integration. Partial least squares (PLS) regression was performed to relate spatial patterns of AI and CFH to regional gene expression. Genes contributing most significantly to the PLS components were subjected to downstream analyses including gene enrichment analysis, protein–protein interaction (PPI) network construction, and spatiotemporal‐specific expression profiling. Spatial correlations were assessed between AI/CFH alterations and neurotransmitter receptor distribution maps, offering insights into potential neurochemical mechanisms linked to functional abnormalities in RRD. Functional alterations in AI and CFH were used as input features for support vector machine (SVM) classification to distinguish RRD patients from controls. SHAP analysis was applied to interpret the contribution of each feature, enhancing model transparency and biological interpretability.

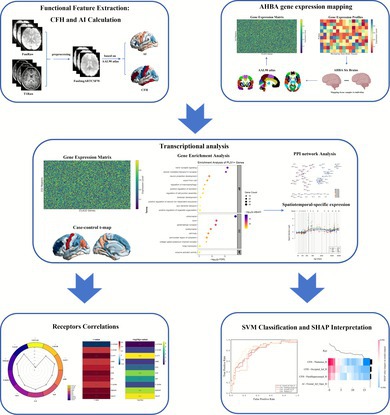

## 1 Introduction

Rhegmatogenous retinal detachment (RRD) is a common and vision‐threatening ophthalmic emergency characterized by the separation of the neurosensory retina from the retinal pigment epithelium due to a full‐thickness retinal break [1]. It is the most prevalent type of retinal detachment, with an estimated annual incidence of approximately 1 per 10,000 individuals in Europe, primarily affecting middle‐aged and elderly populations [2]. Clinically, RRD typically presents with acute onset of photopsia, floaters, and progressive visual field loss, which, if left untreated, can result in permanent vision impairment or even blindness. Despite significant advances in surgical reattachment techniques and high rates of anatomical success, many patients experience unsatisfactory recovery of visual function [3, 4]. This discrepancy suggests that RRD may exert effects beyond retinal damage alone, potentially involving alterations within the central nervous system. Vision is not merely a peripheral sensory function but a complex integrative process dependent on large‐scale brain networks. As an extension of the central nervous system, the retina maintains continuous bidirectional communication with the brain through the retino‐cortical pathway [5]. These considerations have raised growing interest in the potential cerebral consequences of RRD, particularly with respect to cortical plasticity and functional reorganization.

Advances in neuroimaging have recently drawn growing attention to the effects of RRD on the central nervous system, particularly regarding structural and functional changes linked to visual cortical plasticity. Previous studies have identified changes in gray matter volume and functional connectivity in the occipital and temporal lobes, as well as in association cortices of patients with RRD [6, 7, 8]. However, these studies largely emphasize static and large‐scale connectivity patterns, while overlooking fundamental principles of brain organization—namely, regional specialization and interhemispheric integration—both critical for visual processing and complex perception. To address this gap, we apply two emerging voxel‐level metrics—the Autonomy Index (AI) and Connectivity between Functionally Homotopic Voxels (CFH)—to systematically examine RRD‐related functional reorganization. AI measures the difference in functional connectivity between a voxel and its counterparts in both hemispheres, capturing the extent of its functional autonomy or regional specialization [9]. CFH assesses the strength of connectivity between functionally corresponding voxels in opposite hemispheres, reflecting the flexibility and coordination of interhemispheric communication [10]. Together, AI and CFH offer a comprehensive view of brain functional segregation and integration, providing insight into whether retinal damage in RRD leads to widespread functional reorganization. These metrics have shown high sensitivity and clear neurobiological relevance in studies of generalized anxiety disorder [11], Alzheimer's disease [12], and bipolar disorder [13]. These findings highlight their potential for investigating disease mechanisms. Beyond these applications, recent studies have further demonstrated the sensitivity of CFH to interhemispheric dysregulation across diverse neuropsychiatric disorders. For instance, decreased interhemispheric cooperation has been consistently reported in major depressive disorder and schizophrenia, showing strong spatial associations with serotonergic and dopaminergic receptor distributions [14, 15]. Likewise, patients with anxiety disorders exhibit region‐specific CFH alterations, characterized by decreased interhemispheric coupling in occipital and parietal areas and increased coupling in the anterior cingulate cortex, which together suggest impaired hemispheric coordination underlying emotional dysregulation [11]. Collectively, these findings underscore the translational relevance of CFH as a sensitive neural marker of interhemispheric integration, supporting its extension to ophthalmic conditions such as RRD.

Brain function is influenced not only by neural connectivity but also by local molecular characteristics, including gene expression [16, 17, 18] and the spatial distribution of neurotransmitter systems [19, 20, 21]. The Allen Human Brain Atlas (AHBA), the most extensive transcriptomic resource available for the human brain, maps the expression of over 20,000 genes across numerous regions using tissue samples from six healthy adult donors [22, 23]. It serves as a key link between neuroimaging findings and their molecular underpinnings [24]. Integrating AHBA data with neuroimaging provides an opportunity to uncover the molecular basis of large‐scale functional changes, deepening our understanding of brain reorganization. Neurotransmitter systems, which regulate neuronal activity, also play a crucial role in shaping and modulating functional brain networks through their spatial organization. Studies have shown that the functional specialization of brain regions is strongly influenced by the spatial arrangement of neurotransmitter systems [25, 26]. Hansen and colleagues recently combined PET imaging with in vitro binding data from more than 1200 healthy individuals to produce a 3D, standardized atlas of nine major neurotransmitter systems and 19 receptor and transporter types. This atlas offers a valuable neurochemical reference for interpreting the brain's functional architecture [19]. In this study, we integrate AHBA gene expression data with the spatial distribution of neurotransmitter receptors to build a molecular framework that links transcriptomic and neurochemical information, offering new insight into the neurobiological basis of brain reorganization in RRD.

While previous studies have revealed abnormalities in both brain function and molecular profiles in RRD patients, their discriminative potential remains largely unexplored. Traditional statistical methods often fall short in detecting nonlinear or interactive patterns. In contrast, machine learning—particularly pattern recognition—has gained traction in neuroimaging due to its strong performance in classification tasks [27, 28, 29]. To this end, we used a Support Vector Machine (SVM) classifier, a supervised learning algorithm that identifies an optimal decision boundary based on structural risk minimization. SVM is especially suited for neuroimaging applications involving limited sample sizes and high‐dimensional data [30, 31]. To improve the interpretability of conventional “black‐box” models, we incorporated SHapley Additive exPlanations (SHAP). SHAP quantifies each feature's contribution to the classification outcome, enabling simultaneous evaluation of model performance and interpretability [32]. Through this interpretable machine learning framework, we evaluated the potential discriminative utility of AI and CFH in detecting functional abnormalities in RRD, while identifying key brain regions and their contributions—offering valuable insight for the development of biologically meaningful imaging indicators.

**FIGURE 1:**
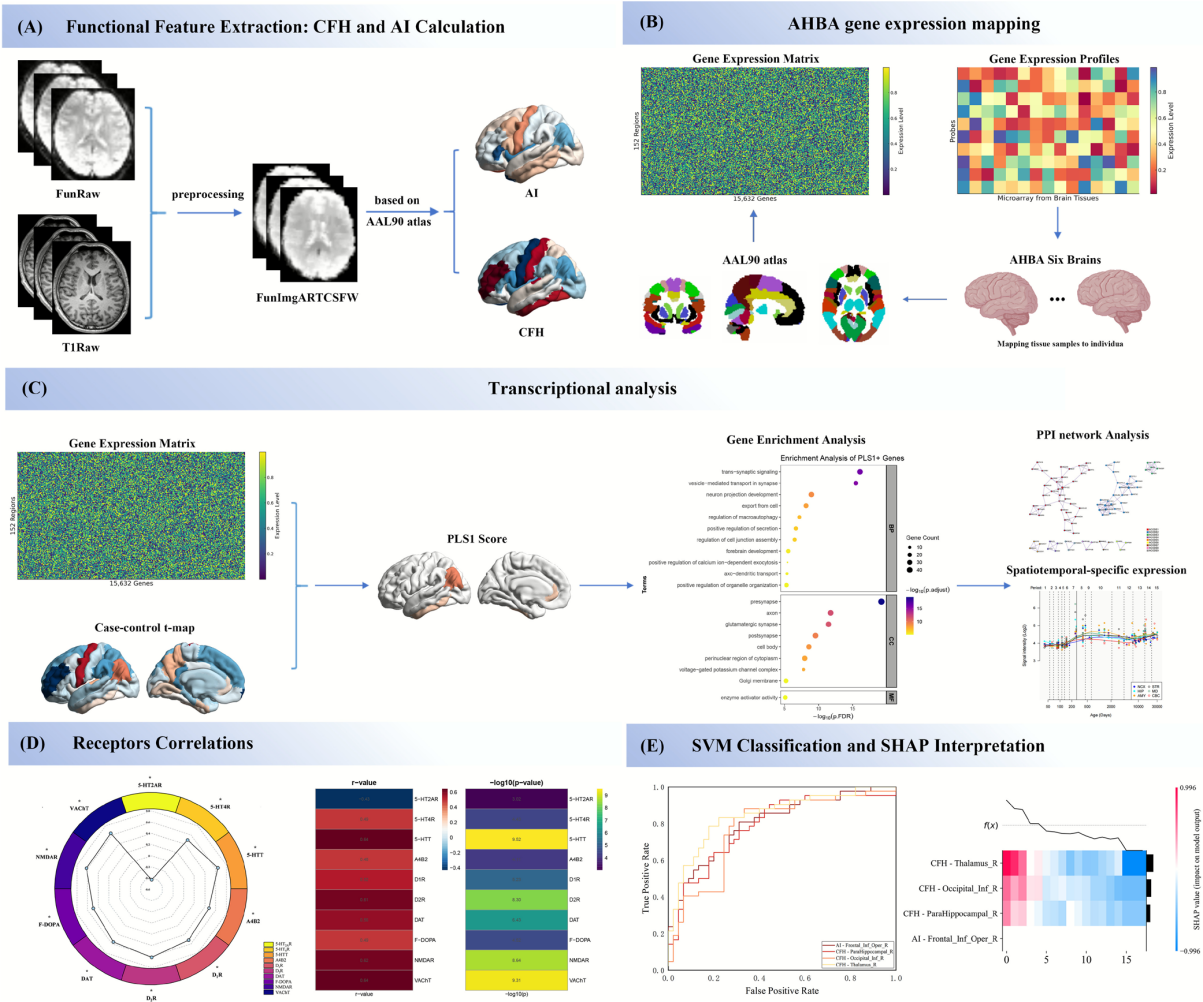
Overview of the study design. (A) Calculation of AI and CFH. Based on the AAL90 atlas, AI and CFH values were computed for each RRD patient. (B) Gene expression data. A gene expression matrix was constructed by extracting the expression values of all genes from the left hemisphere regions using the AHBA. (C) Transcriptomic analysis. PLS regression was performed to associate AI and CFH values with regional gene expression. Genes strongly contributing to the PLS were subjected to enrichment analysis, PPI network analysis, and spatiotemporal specificity expression profiling. (D) Receptor correlation analysis. Spatial correlations between AI and CFH alterations and neurotransmitter receptor distributions were examined. (E) SVM and SHAP analysis. The signal values from regions showing significant AI and CFH differences were used as features for SVM classification and SHAP analysis. AI, autonomy index; AHBA, Allen Human Brain Atlas; CFH, connectivity between functionally homotopic voxels; PLS, partial least squares; PPI, protein–protein interaction; SHAP, Shapley additive explanations; SVM, support vector machine.

We hypothesize that patients with RRD show marked disruptions in whole‐brain functional autonomy and interhemispheric coordination. These abnormalities may follow distinct spatial patterns and be linked to specific gene expression profiles and the spatial distribution of neurotransmitter systems. To test this hypothesis, we used two voxel‐level metrics—AI and CFH—to assess brain functional organization in terms of regional specialization and interhemispheric coordination in RRD patients. We then integrated data from the AHBA and neurotransmitter receptor density maps to investigate the molecular mechanisms driving these functional changes. Finally, we applied an SVM combined with SHAP analysis to assess the discriminative potential of these metrics and identify key contributing features. An overview of the study design and analytical workflow is shown in Figure 1.

## 2 Participants and Methods

### 2.1 Participants

This study protocol was approved by the Institutional Review Board of the First Affiliated Hospital of Nanchang University, and all participants provided written informed consent prior to enrollment. A total of 87 individuals participated in the study, including 42 patients diagnosed with RRD and 45 age‐, sex‐, and education‐matched healthy controls (HCs) recruited from the local community.

RRD was diagnosed based on comprehensive ophthalmic examinations—including fundus evaluation, optical coherence tomography (OCT), and B‐scan ultrasonography—in accordance with standard clinical guidelines. All diagnoses were jointly confirmed by two experienced retinal specialists from the Department of Ophthalmology at the First Affiliated Hospital of Nanchang University.

Inclusion criteria for the RRD group were as follows: (1) spontaneous retinal detachment with at least one identifiable retinal break; (2) involvement of one or more retinal quadrants; and (3) absence of coexisting ocular pathologies in either eye. Exclusion criteria included secondary causes of RRD, such as high myopia‐associated detachment, traumatic detachment, diabetic retinopathy, prior vitreoretinal surgery, as well as systemic conditions such as cardiovascular disease, neuropsychiatric disorders, or cerebrovascular events.

All HCs underwent detailed ophthalmological and neurological screening to confirm: (1) no history of ocular or systemic disease; (2) best‐corrected visual acuity (BCVA) of ≥ 1.0; and (3) normal findings on magnetic resonance imaging (MRI), OCT, and B‐scan ultrasonography.

A post hoc power analysis was conducted using G*Power version 3.1.9.7 [33] to evaluate the adequacy of the sample size. Assuming a two‐tailed independent‐samples *t*‐test with an alpha level of 0.05 and a medium effect size (Cohen's *d* = 0.5), the achieved statistical power (1–β) for the present sample (RRD = 42, HC = 45) was 0.6346, indicating moderate sensitivity for detecting between‐group differences.

Demographic characteristics, including age, sex, and education level, were statistically matched between the two groups to ensure comparability.

### 2.2 fMRI Data Acquisition

Functional MRI data were acquired using a 3.0T Siemens Trio Tim scanner (Siemens Medical Solutions, Erlangen, Germany) equipped with an 8‐channel phased‐array head coil at the First Affiliated Hospital of Nanchang University. During scanning, participants were instructed to lie in the supine position with their eyes closed, remain awake, stay relaxed, and avoid engaging in any specific cognitive activity. A sleep‐monitoring system was employed to ensure participants remained alert throughout the procedure. To minimize scanner noise and head motion, earplugs and foam padding were used to stabilize the head and reduce acoustic interference. Detailed imaging parameters are summarized in Table 1.

**TABLE 1:** Scanning parameters for BOLD sequences and structural T1‐weighted images.

Scanning parameters	3D‐T1	EPI
TR (ms)	1900	2000
TE (ms)	2.26	30
FOV (mm^2^)	256 × 256	200 × 200
matrix	256 × 256	64 × 64
Slice thickness (mm)	1	4
Interslice gap (mm)	0.5	1.2

Abbreviations: FOV, field of view; TE, echo time; TR, repetition time.

### 2.3 Data Preprocessing

Structural and functional MRI data were preprocessed using the WhiteMatterSF Toolbox (https://github.com/jigongjun/Neuroimaging‐and‐Neuromodulation), which integrates relevant functional modules from SPM12 (https://www.fil.ion.ucl.ac.uk/spm/software/spm12/) and FSL (https://fsl.fmrib.ox.ac.uk/fsl/docs/#/). T1‐weighted structural images were converted to NIfTI format, segmented into gray matter (GM), white matter (WM), and cerebrospinal fluid (CSF), and then subjected to quality control. Functional image preprocessing followed a standard resting‐state fMRI pipeline, including the following steps: (1) Conversion to NIfTI format; (2) Removal of the first 10 time points to reduce signal instability; (3) Slice‐timing correction; (4) Head motion correction; (5) Coregistration with the structural image; (6) Regression of nuisance variables, including 24 head motion parameters and mean signals from the whole brain, WM, and CSF; (7) Spatial smoothing using a 4 mm FWHM Gaussian kernel; (8) Temporal bandpass filtering (0.01–0.1 Hz); (9) Spatial normalization to the MNI template space. All preprocessing steps conformed to standard practices widely used in resting‐state fMRI research.

### 2.4 AI and CFH Calculation

AI quantifies the degree to which a given voxel exhibits preferential functional connectivity with ipsilateral rather than contralateral regions, thereby serving as a proxy for functional specialization [9, 34]. AI was calculated using the following formula:

$$\mathrm{AI}=\left(\mathrm{Ni}/\mathrm{Hi}\right)-\left(\mathrm{Nc}/\mathrm{Hc}\right)$$

where ‘Ni’ and ‘Nc’ represent the number of significantly connected voxels within the ipsilateral and contralateral hemispheres, respectively, and ‘Hi’ and ‘Hc’ denote the total number of voxels in the corresponding hemispheres. A whole‐brain AI map was generated for each participant.

CFH is a voxel‐wise metric that quantifies interhemispheric cooperation by measuring the functional similarity between mirrored regions across hemispheres [10, 15]. The calculation proceeded as follows:

1. for each voxel, whole‐brain functional connectivity (FC) was computed;
2. in the opposite hemisphere, the voxel with the highest FC value was identified and designated as the “functionally homotopic voxel”;
3. the CFH value was defined as the Pearson correlation coefficient between the original voxel and its homotopic counterpart;
4. resulting CFH values were normalized across the whole brain to improve normality and comparability.

Voxel‐wise CFH maps were then generated for all participants. Higher CFH values indicate greater interhemispheric communication.

### 2.5 Calculation of Regional Gene Expression

Regional gene expression data were obtained from the AHBA (http://human.brain‐map.org), which contains microarray‐based expression measurements from 3702 brain tissue samples across six neurotypical adult donors [22]. Detailed sample information is provided in Table S1.

Preprocessing of the AHBA dataset was performed using the Python‐based “abagen” toolbox (https://github.com/rmarkello/abagen), following standardized protocols [35]. The following steps were applied:

1. probe‐to‐gene annotation using updated genome mappings;
2. exclusion of low‐quality probes whose expression values fell below background noise in > 50% of samples;
3. selection of a single representative probe per gene based on maximum inter‐regional expression consistency;
4. assignment of tissue samples to the nearest region within the AAL90 atlas using a 2‐mm Euclidean distance threshold;
5. normalization of expression values across samples using a scaled robust sigmoid function.

Given that only two donors included data from the right hemisphere, analyses were restricted to the left hemisphere. The final output was a regional expression matrix comprising 45 left‐hemisphere brain regions and 15,633 genes.

### 2.6 Transcriptomic Correlates of AI and CFH Alterations

Partial least squares (PLS) regression [36] was employed to examine the association between regional gene expression patterns and AI/CFH alterations, represented by t‐values derived from group comparisons (RRD vs. HCs) across 45 left‐hemisphere brain regions. The first PLS component (PLS1) captures the linear combination of gene expression variables most strongly correlated with the observed pattern of functional alterations. To assess whether the covariance explained by PLS1 exceeded chance levels, a nonparametric permutation test (5000 iterations) was conducted. Subsequently, the contribution of individual genes to PLS1 was evaluated using a bootstrap resampling procedure (10,000 iterations), generating standard errors for each gene's loading. Z‐scores were calculated as the ratio between each gene's loading and its bootstrap‐estimated standard error, enabling genes to be ranked based on their relative contribution to PLS1 [37, 38]. Genes with Z values corresponding to the false discovery rate (FDR)‐corrected *p* < 0.05 were defined as significantly contributing to PLS1, and were further categorized into PLS1+ and PLS1− gene sets based on the direction of their weights. The lists of significant PLS1+ and PLS1− genes are provided in Table S2.

### 2.7 Enrichment Analysis

To further elucidate the biological significance of the PLS1+ and PLS1− gene sets, functional enrichment analyses were conducted using the Metascape platform (https://metascape.org) [39]. Gene Ontology (GO) terms and Kyoto Encyclopedia of Genes and Genomes (KEGG) pathways were used to identify overrepresented biological processes, molecular functions, and cellular components. Enrichment results were considered statistically significant at a threshold of *p* < 0.05, corrected for multiple comparisons using the FDR method. All analyses were performed separately for the PLS1+ and PLS1− gene sets.

### 2.8 Protein–Protein Interaction Network Analysis

To explore potential functional interactions at the protein level, protein–protein interaction (PPI) networks were constructed for genes significantly contributing to PLS1 using the Metascape platform (https://metascape.org) [39]. The resulting networks were visualized using Cytoscape software [40]. This analysis allowed the identification of tightly connected subnetworks and potential hub proteins, providing insight into key biological pathways and processes associated with the transcriptomic signatures of AI and CFH alterations.

### 2.9 Temporal‐Specific Expression Analysis

To investigate the developmental timing of gene expression associated with AI and CFH alterations, temporal expression profiles of representative genes from the PLS1+ and PLS1− sets were examined using the Human Brain Transcriptome (HBT) database (https://hbatlas.org/pages/hbtd). Genes with the highest absolute Z‐scores in each set were selected to represent the most strongly weighted transcriptomic features. The relative expression levels of these genes across multiple developmental stages were analyzed to identify temporal enrichment patterns, reflecting whether a given gene exhibited preferential expression during specific life periods. This analysis allowed for the characterization of stage‐specific transcriptomic signatures potentially linked to neurodevelopmental processes underlying RRD‐related brain reorganization.

### 2.10 Spatial Correlation With Neurotransmitter Receptor and Transporter Density

To investigate the potential neurochemical substrates underlying regional alterations in AI and CFH in RRD patients, we examined their spatial correspondence with neurotransmitter receptor and transporter densities derived from in vivo molecular imaging studies. A total of 39 positron emission tomography (PET)‐based whole‐brain receptor density maps were obtained from publicly available datasets [41] (https://github.com/netneurolab/hansen_receptors/tree/main). Receptor density values were extracted and averaged within 90 cortical regions defined by the AAL90 atlas. Similarly, voxelwise t‐values representing between‐group differences (RRD vs. HCs) in AI and CFH were computed using two‐sample *t*‐tests and then averaged across the same parcellation scheme. Spearman correlation analyses were performed between the regional AI/CFH t‐maps and receptor density distributions to assess spatial similarity. To account for intrinsic spatial autocorrelation, a nonparametric spin‐based permutation test (5000 iterations) was employed to generate a spatial null distribution of correlation coefficients. Statistical significance was determined by comparing the empirical correlation to this null distribution, with a two‐tailed threshold of *p* < 0.001 corrected using Bonferroni adjustment for multiple comparisons.

### 2.11 SVM and SHAP Analysis

To assess the discriminative power of AI and CFH features in distinguishing RRD patients from HCs, a machine learning classification framework was implemented based on SVM modeling. The analysis was performed using MATLAB 2017b, incorporating the libsvm‐3.24 library and custom svm_function scripts [42]. A radial basis function (RBF) kernel was employed to enable nonlinear separation, and hyperparameters were optimized via grid search to determine the optimal configuration of the decision boundary [43]. Model performance was evaluated using leave‐one‐out cross‐validation (LOOCV), in which each subject was iteratively used as a test case while the remaining subjects formed the training set. Classification performance was quantified using multiple metrics, including accuracy, sensitivity, specificity, precision, and area under the ROC curve (AUC).

To improve model interpretability and identify key predictive features, SHAP was applied in Python. SHAP, based on cooperative game theory, assigns additive importance values to each feature by estimating its marginal contribution to model predictions. This approach enables both global and subject‐level insights into feature relevance, transforming the SVM from a black‐box classifier into an interpretable decision framework.

### 2.12 Correlation Analysis

To investigate the potential associations between neuroimaging indicators and clinical characteristics, Spearman's rank correlation analysis was employed. Clinical variables included two key parameters: visual acuity and disease duration. Neuroimaging indicators comprised the AI and CFH, both of which exhibited significant abnormalities in patients with RRD. Since the clinical variables did not follow a normal distribution, the Spearman correlation coefficient (ρ) was used to evaluate the monotonic relationships between imaging features and clinical measures. All statistical analyses were conducted using SPSS version 27.0 (SPSS Inc., Chicago, IL, USA), and a two‐tailed *p* < 0.05 was considered statistically significant.

### 2.13 Statistical Analysis

The normality of demographic and clinical variables was tested using the Shapiro–Wilk test. Variables that followed a normal distribution were analyzed using independent‐samples *t*‐tests, while non‐normally distributed variables were compared using the Mann–Whitney *U* test. Categorical variables were examined using the chi‐square test. Between‐group comparisons of demographic and clinical variables were performed in SPSS version 27. Group‐level differences in AI and CFH metrics were assessed using voxel‐wise two‐sample *t*‐tests, with multiple comparisons corrected by Gaussian random field (GRF) theory at a voxel‐level threshold of *p* < 0.001 and a cluster‐level threshold of *p* < 0.05 (two‐tailed). To control for potential confounding effects, age, sex, and years of education were included as covariates in all imaging analyses. These adjustments were made to enhance the validity and robustness of the observed results.

## 3 Results

### 3.1 Demographic Characteristics

No significant differences in gender and age were observed between the two groups of patients. Detailed information is provided in Table 2.

**TABLE 2:** Demographic and Clinical Features of RRD and HCs.

	RRD	HCs	t value	*p*
Men/women	18/24	18/27	0.073	0.787^x^
Age (years, median (Q1, Q3))	52.5 (42.75, 67.5)	54 (45.5, 60.5)	0.500	0.618^t^
Duration of detachment (days, median (Q1, Q3))	11 (7, 30)	N/A	N/A	N/A
IOP (mmHg, median (Q1, Q3))	15 (12, 17)	N/A	N/A	N/A
Vision (mean, median (Q1, Q3))	0.040 (0.010, 0.128)	N/A	N/A	N/A
Axial length of eye (mm, mean ± STD)	24.702 ± 2.150	N/A	N/A	N/A
HAMA score (median (Q1, Q3))	3.5 (2, 6)	N/A	N/A	N/A

Abbreviations: HAMA, Hamilton Anxiety Scale; HCs, healthy controls; IOP, intraocular pressure; N/A, not applicable; Q1, first quartile (25th percentile); Q3, third quartile (75th percentile); RRD, rhegmatogenous retinal detachment; STD, standard deviation; ^t^, independent samples *t*‐test; ^x^, chi‐square test.

### 3.2 Group Differences in Hemispheric Specialization and Interhemispheric Cooperation

**FIGURE 2:**
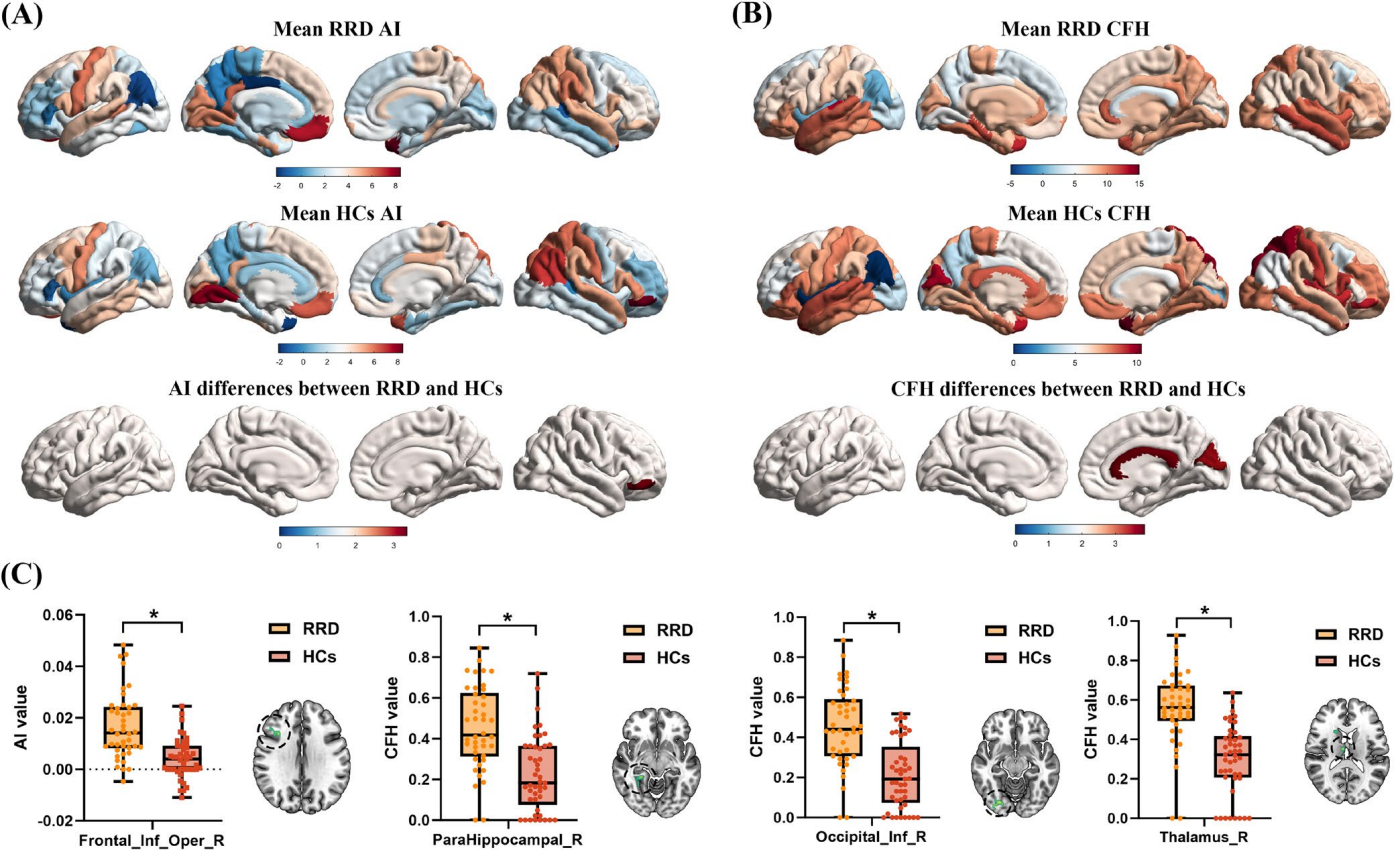
Group differences in hemispheric specialization and interhemispheric cooperation between RRD patients and HCs. (A) Hemispheric specialization maps for the RRD and HCs derived from one‐sample *t*‐tests, and the between‐group contrast based on two‐sample *t*‐tests. RRD patients showed significantly increased AI in the Frontal_Inf_Oper_R. (B) Interhemispheric cooperation maps for each group and their between‐group contrast. RRD patients exhibited significantly higher CFH values in the ParaHippocampal_R, Occipital_Inf_R, and Thalamus_R. (C) Bar plots depicting significant between‐group differences in AI and CFH values across the positive regions. Statistical threshold: GRF correction: Voxel‐level *p* < 0.001, cluster‐level *p* < 0.05, two‐tailed. *, significant; AI, autonomy index; CFH, connectivity between functionally homotopic voxels; Frontal_Inf_Oper_R, right inferior frontal gyrus, opercular part; HCs, healthy controls; ParaHippocampal_R, right parahippocampal gyrus; Occipital_Inf_R, right inferior occipital gyrus; RRD, rhegmatogenous retinal detachment; Thalamus_R, right thalamus.

Figure 2A presents the hemispheric specialization patterns derived from one‐sample *t*‐tests for the RRD and HC groups, as well as between‐group differences based on two‐sample *t*‐tests. Compared to HCs, RRD patients exhibited significantly increased AI values in the right inferior frontal gyrus, opercular part (Frontal_Inf_Oper_R) (GRF corrected: voxel‐level *p* < 0.001, cluster‐level *p* < 0.05, two‐tailed).

Figure 2B illustrates interhemispheric cooperation patterns within each group and the corresponding between‐group comparisons. RRD patients showed significantly elevated CFH values in the right parahippocampal gyrus (ParaHippocampal_R), right inferior occipital gyrus (Occipital_Inf_R), and right thalamus (Thalamus_R) (GRF corrected: voxel‐level *p* < 0.001, cluster‐level *p* < 0.05, two‐tailed).

Figure 2C and Table 3 summarize the regions showing significant between‐group differences in AI and CFH metrics. Specifically, AI was significantly higher in RRD patients in Frontal_Inf_Oper_R, while CFH values were significantly increased in ParaHippocampal_R, Occipital_Inf_R, and Thalamus_R relative to HCs.

**TABLE 3:** Brain regions with altered hemispheric specialization and interhemispheric cooperation in RRD patients compared to HCs.

	Brain regions	Peak t‐score	MNI coordinates (x, y, z)	Cluster size (voxels)
AI	Frontal_Inf_Oper_R	4.642	33, 18, 30	42
CFH	ParaHippocampal_R	5.848	24, −39, −9	66
	Occipital_Inf_R	4.963	33, −84, −12	57
	Thalamus_R	5.801	6, −12, 15	159

*Note:* GRF correction: voxel‐level *p* < 0.001, cluster‐level *p* < 0.05, two‐tailed.

Abbreviations: AI, autonomy index; CFH, connectivity between functionally homotopic voxels; Frontal_Inf_Oper_R, right inferior frontal gyrus, opercular part; HCs, healthy controls; MNI, montreal neurological institute; Occipital_Inf_R, right inferior occipital gyrus; ParaHippocampal_R, right parahippocampal gyrus; RRD, rhegmatogenous retinal detachment; Thalamus_R, right thalamus.

### 3.3 Transcription–Neuroimaging Associations

**FIGURE 3:**
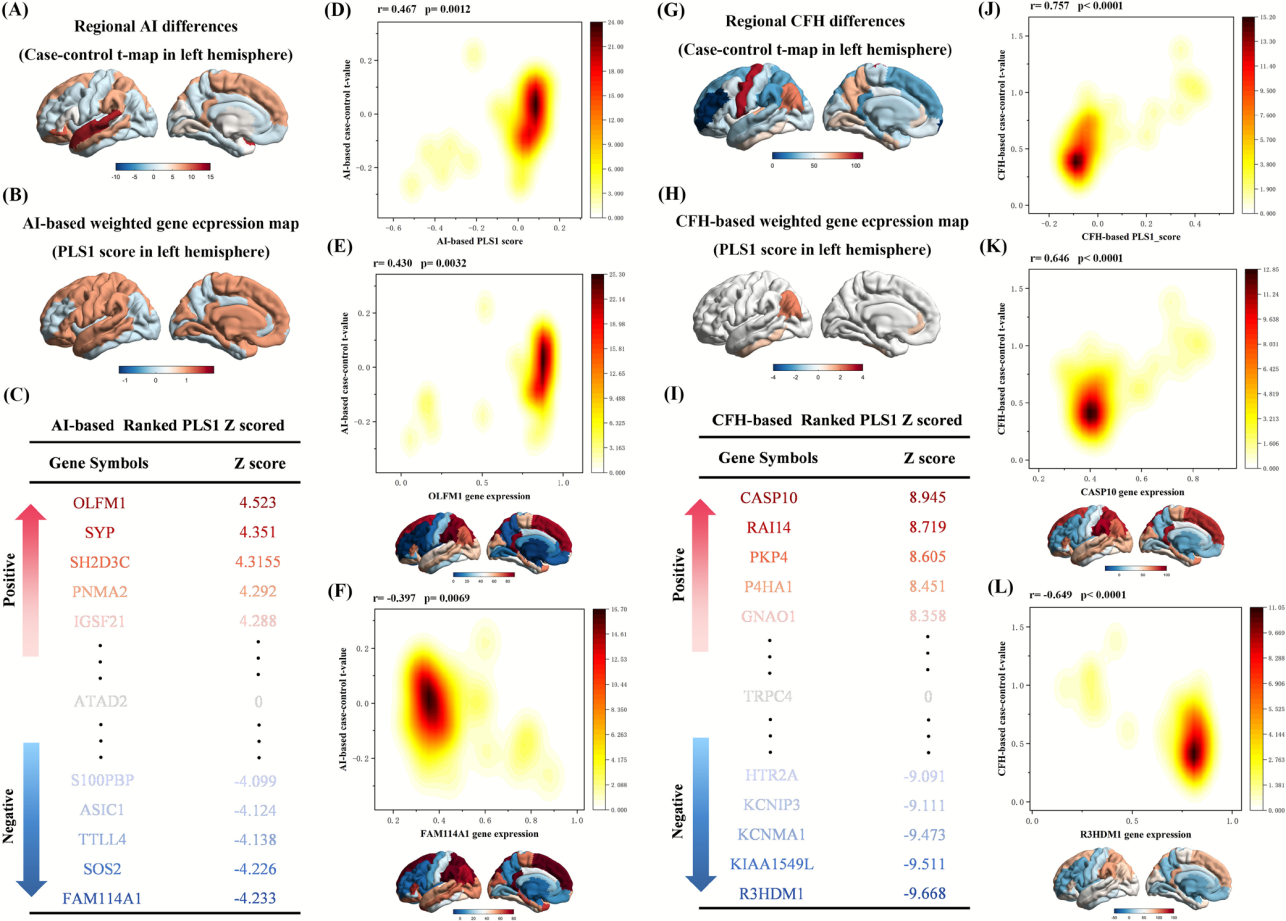
Transcriptomic associations with altered hemispheric specialization and interhemispheric cooperation in RRD patients. (A)–(F) represent results from the AI‐based partial least squares (PLS) analysis: (A) Group difference t‐map of regional AI values in the left hemisphere. (B) AI‐based weighted gene expression map derived from regional PLS1 scores. (C) Ranked AI‐associated genes based on z‐scored PLS1 weights. (D) Scatter plot with density overlay showing the correlation between regional PLS1 scores and the AI group difference map (*r* = 0.467, *p* = 0.0012). (E) Correlation of representative positively weighted gene (OLFM1) with the AI t‐map (*r* = 0.430, *p* = 0.0032). (F) Correlation of representative negatively weighted gene (FAM114A1) with the AI t‐map (*r* = −0.397, *p* = 0.0069). (G)–(L) represent results from the CFH‐based PLS analysis: (G) Group difference t‐map of regional CFH values in the left hemisphere. (H) CFH‐based weighted gene expression map derived from regional PLS1 scores. (I) Ranked CFH‐associated genes based on z‐scored PLS1 weights. (J) Scatter plot with density overlay showing the correlation between regional PLS1 scores and the CFH t‐map (*r* = 0.757, *p* < 0.0001). (K) Correlation of representative positively weighted gene (CASP10) with the CFH t‐map (*r* = 0.646, *p* < 0.0001). (L) Correlation of representative negatively weighted gene (R3HDM1) with the CFH t‐map (*r* = −0.649, *p* < 0.0001). AI, autonomy index; CFH, connectivity between functionally homotopic voxels; PLS, partial least squares; PLS1, first PLS component; RRD, rhegmatogenous retinal detachment.

To identify transcriptional features corresponding to the spatial distribution of AI and CFH alterations, PLS regression was applied to link regional gene expression profiles with the voxelwise t‐maps of group differences between RRD patients and HCs (Figure 3A,G).

For AI, the PLS1 explained 52.42% of the variance in the AI difference map, significantly exceeding the null distribution derived from spatial permutation testing (spin test, *p* < 0.005, *r* = 0.5498). The AI‐based PLS1 score map revealed a weighted gene expression gradient across the left hemisphere (Figure 3B). Following established procedures [37, 44], 481 positively weighted (PLS1+) genes (*Z* > 3.05) and 230 negatively weighted (PLS1−) genes (*Z* < −3.05) were identified based on standardized loading scores and an FDR‐corrected significance threshold (*p* < 0.05) (Figure 3C). The AI‐based PLS1 expression map was significantly correlated with the AI group‐difference t‐map (*r* = 0.467, *p* = 0.0012; Figure 3D). Representative genes with the highest and lowest PLS1 weights are illustrated in Figure 3E,F, respectively. Full gene lists, along with Z‐scores and locations, are provided in Table S2.

Similarly, in the CFH analysis, PLS1 explained 59.23% of the variance in the CFH t‐map, again exceeding chance levels (spin test, *p* < 0.005, *r* = 0.7092). The CFH‐based PLS1 score map also revealed a spatial gene expression gradient across the left hemisphere (Figure 3H). Based on the same criteria, 4523 PLS1+ genes (*Z* > 2.20) and 4163 PLS1− genes (*Z* < −2.20) were identified (Figure 3I). The CFH‐based PLS1 expression pattern was strongly correlated with the CFH t‐map (*r* = 0.757, *p* < 0.0001; Figure 3J). Examples of representative high‐ and low‐weighted genes are shown in Figure 3K,L, and detailed gene information is available in Table S2.

### 3.4 Enrichment Pathways of Genes Associated With Changes in AI/CFH

**FIGURE 4:**
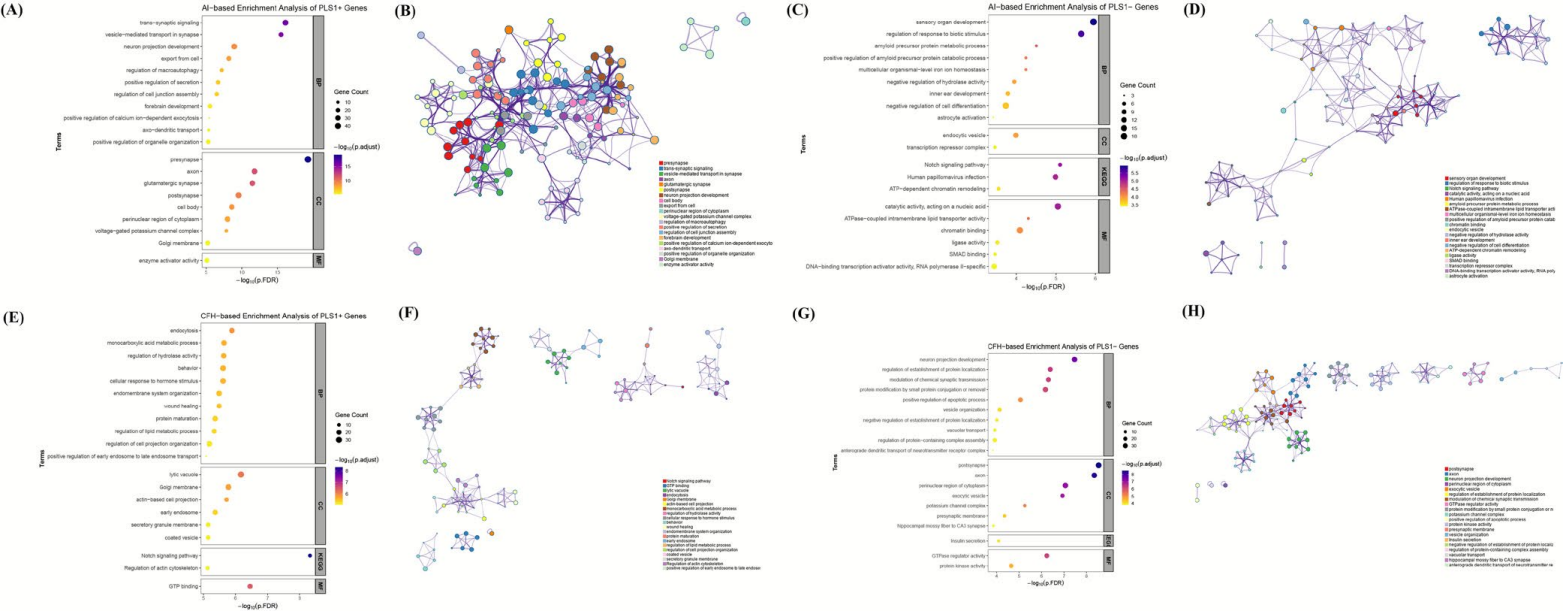
Functional enrichment and network analyses of genes associated with AI and CFH alterations. (A–D) Analyses based on AI‐associated gene sets. (A) GO and KEGG enrichment analysis of PLS1^+^ genes showing significant enrichment in biological processes related to trans‐synaptic signaling, neuron projection development, and vesicle‐mediated transport. (B) Metascape enrichment network visualization showing the intracluster and intercluster similarities of enriched pathways for AI‐based PLS1^+^ genes. Each pathway is represented by a node, with node size corresponding to the number of input genes, and color denoting different functional clusters. (C) GO and KEGG enrichment analysis of PLS1^−^ genes highlighting biological processes related to sensory organ development, transcriptional regulation, and chromatin remodeling. (D) Metascape enrichment network visualization showing the functional relationships among enriched terms for AI‐based PLS1^−^ genes. Node size represents the number of genes in each pathway, and colors indicate distinct enrichment clusters. (E–H) Analyses based on CFH‐associated gene sets. GO and KEGG enrichment analysis of PLS1^+^ genes showing enrichment in endomembrane organization, lipid metabolism, and Notch signaling pathway. (F) Metascape enrichment network visualization displaying the interrelationships of enriched biological pathways for CFH‐based PLS1^+^ genes, with node size proportional to gene count and colors indicating cluster membership. (G) GO and KEGG enrichment analysis of PLS1^−^ genes demonstrating enrichment in synaptic signaling, axonogenesis, and apoptotic regulation. (H) Metascape enrichment network visualization of CFH‐based PLS1^−^ genes showing the functional connectivity among synaptic, cytoskeletal, and regulatory pathways. Node size corresponds to pathway gene number, and color represents functional modules. AI, autonomy index; BP, biological process; CC, cellular component; CFH, connectivity between functionally homotopic voxels; FDR, false discovery rate; GO, Gene Ontology; KEGG, Kyoto Encyclopedia of Genes and Genomes; MCODE, molecular complex detection; MF, molecular function; PLS, partial least squares; PLS1, first PLS component.

To characterize the biological functions of genes associated with regional alterations in AI and CFH, we performed functional annotation using Metascape. GO and KEGG enrichment analyses revealed both overlapping and distinct biological processes for AI‐ and CFH‐associated gene sets. For the AI‐based PLS1+ gene set, enrichment was observed for GO terms related to trans‐synaptic signaling and presynaptic structure (Figure 4A,B), indicating a strong involvement in synaptic communication and neuronal transmission. In contrast, the AI‐based PLS1− genes were significantly enriched in pathways related to sensory organ development and the regulation of responses to biotic stimuli (Figure 4C,D), suggesting roles in broader neurodevelopmental and environmental adaptation processes. For the CFH‐based PLS1+ genes, KEGG analysis revealed significant enrichment in the Notch signaling pathway (Figure 4E,F), implicating this gene set in cell fate determination and neurogenesis. Meanwhile, CFH‐based PLS1− genes were highly enriched for GO terms such as postsynapse and axon (Figure 4G,H), highlighting their relevance to synaptic organization and axonal structure. These results suggest that AI and CFH alterations may be underpinned by partially distinct molecular mechanisms, with AI‐related genes more strongly linked to synaptic signaling and CFH‐related genes to developmental and structural plasticity.

### 3.5 PPI Network Analysis of Genes Associated With Changes in AI and CFH

PPI networks were constructed for PLS1^+^ and PLS1^−^ gene sets derived from AI‐ and CFH‐related analyses. Using the Metascape platform with MCODE clustering, we identified putative functional modules and underlying biological mechanisms.

In the AI‐associated network, the PLS1^+^ genes formed nine distinct functional clusters (Figure S1A). The top‐ranked module (MCODE1) was enriched for proteins involved in synaptic vesicle cycling and endocytosis, suggesting a role in synaptic signaling and membrane trafficking. In contrast, the PLS1^−^ gene network (Figure S1B) yielded two clusters. MCODE1 was associated with intracellular signaling, epigenetic regulation, and protein transport, while MCODE2 was centered on HDAC1 and REST, implicating transcriptional repression and chromatin remodeling.

For the CFH‐associated network, the PLS1^+^ gene set also formed nine functional modules (Figure S1C). MCODE1 was predominantly related to mitochondrial metabolism and cellular stress response, whereas MCODE2 involved epigenetic regulators. Other modules were linked to cytoskeletal organization, energy metabolism, and transcriptional activity. The CFH‐based PLS1^−^ network (Figure S1D) revealed 10 modules. MCODE1 and MCODE2 were enriched for synaptic and autophagy‐related proteins, respectively, while MCODE5 included genes involved in RNA splicing, and MCODE10 featured motor proteins, suggesting roles in cellular homeostasis, gene regulation, and axonal transport.

Together, these results indicate that the molecular architecture underlying AI and CFH alterations is modular, with distinct biological pathways implicated in synaptic function, transcriptional control, and cellular structural dynamics.

### 3.6 Temporal‐Specific Expression Analysis of Representative Genes

To further elucidate the potential developmental roles of key genes identified through PLS models, we examined the spatiotemporal expression trajectories of the top‐weighted representative genes (based on Z‐scores) in both PLS1^+^ and PLS1^−^ components for AI and CFH. The analysis covered 15 developmental periods across six brain regions. As shown in Figure S2A, OLFM1, a representative gene from the AI‐based PLS1^+^ component, exhibited a pronounced spatiotemporal expression profile, with the highest expression levels observed in the neocortex (NCX) and hippocampus (HIP). Its expression increased rapidly after birth, peaked during adolescence, and remained stable thereafter, suggesting a critical role in cortical maturation and cognitive development. In contrast, its expression in the cerebellum (CBC) remained consistently low and declined with age, indicating strong regional specificity. Overall, OLFM1 followed an “early upregulation–stable plateau–slight decline” trajectory, supporting its involvement in early and mid‐stage neurodevelopment. Figure S2B illustrates the expression pattern of FAM114A1, a representative gene from the AI‐based PLS1^−^ component. This gene showed moderately elevated expression in early neocortical stages (Periods 1–2), followed by a rapid decline and stabilization. Expression levels remained moderate in the hippocampus and amygdala, with relatively low temporal variability, indicating a temporally stable expression profile. These findings suggest a possible role for FAM114A1 in long‐term modulatory processes or cell type–specific functions within the nervous system. For the CFH‐based PLS1^+^ component, CASP10 demonstrated a low but broad expression pattern across brain regions (Figure S2C). Expression slightly increased during early postnatal development (Period 8), reached a plateau, and showed a modest rebound after adolescence. The absence of pronounced regional specificity suggests a generalized function across brain areas. As a gene involved in apoptosis, the temporal dynamics of CASP10 expression may relate to early neurodevelopmental processes regulating cell survival and homeostasis. Finally, Figure S2D shows the trajectory of R3HDM1, a representative gene from the CFH‐based PLS1^−^ component. This gene displayed a sharp upregulation around the perinatal period (Periods 6–8), followed by sustained high‐level expression in most brain regions, especially within the neocortex. Its expression remained low in the cerebellum, reflecting notable regional specificity. The “early upregulation–stable maintenance” profile suggests its potential involvement in cortical maturation and long‐term neural network stabilization.

### 3.7 Associations Between AI/CFH Abnormalities and Neurotransmitter Receptor/Transporter Density

**FIGURE 5:**
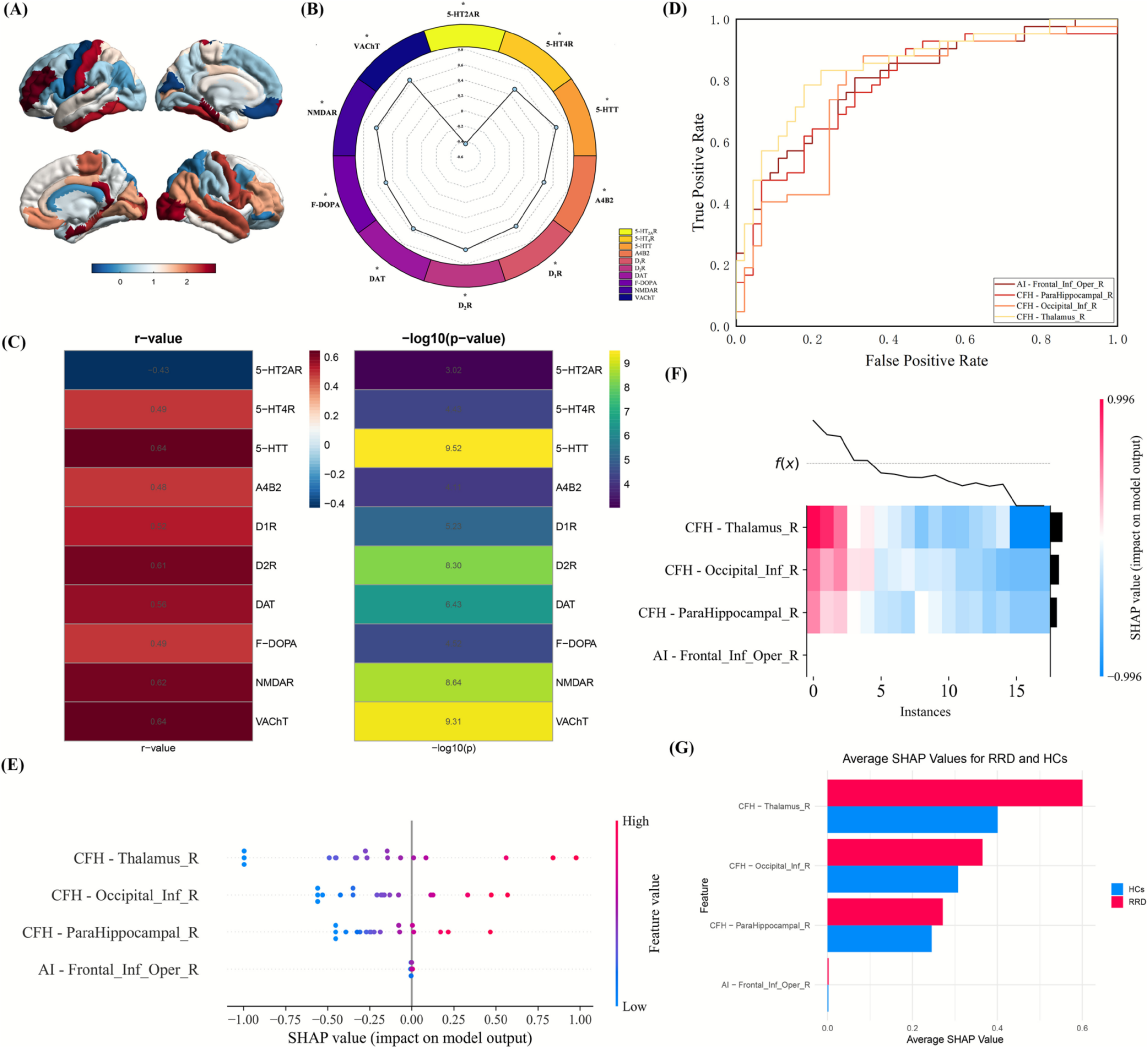
Spatial associations between AI/CFH alterations and neurotransmitter receptor/transporter densities, and SVM‐based classification with SHAP interpretation. (A) Surface rendering of CFH‐related t‐values showing interhemispheric cooperation differences between RRD patients and HCs, mapped onto 3D brain templates. (B) Polar plot visualizing the strength of correlations between CFH alterations and ten neurotransmitter systems. (C) Left: Heatmap of Spearman correlation coefficients (r‐values) between CFH t‐maps and neurotransmitter receptor/transporter densities; Right: Corresponding –log_10_(*p*) values. Significant correlations (Bonferroni‐corrected *p* < 0.001) were observed for 5‐HT_2_AR, 5‐HT_4_R, 5‐HTT, A4B2, D_1_R, D_2_R, DAT, F‐DOPA, NMDAR, and VAChT. (D) ROC curves of SVM classifiers based on regional AI/CFH values, showing discriminative performance in distinguishing RRD patients from HCs. (E) SHAP summary dot plot indicating the relative importance of individual brain regions in model prediction. Each point represents a subject's SHAP value for a given feature. (F) SHAP decision plot depicting the impact of the top four brain regions on the SVM model output across individuals. (G) Bar plot of average SHAP values for each region, stratified by group. CFH values in the right thalamus, occipital gyrus, and parahippocampal gyrus, as well as AI values in the right inferior frontal gyrus (opercular part), were the most influential predictors. 5‐HT_2A_R, 5‐Hydroxytryptamine 2A receptor; 5‐HT_4_R, 5‐Hydroxytryptamine 4 receptor; 5‐HTT, 5‐Hydroxytryptamine transporter; A4B2, Alpha4Beta2 nicotinic acetylcholine receptor; AI, autonomy index; CFH, connectivity between functionally homotopic voxels; D_1_R, dopamine D1 receptor; D_2_R, dopamine D2 receptor; DAT, dopamine transporter; F‐DOPA, 6‐[^18^F]fluoro‐L‐DOPA uptake; Frontal_Inf_Oper_R, right inferior frontal gyrus, opercular part; NMDAR, N‐Methyl‐D‐aspartate receptor; Occipital_Inf_R, right inferior occipital gyrus; ParaHippocampal_R, right parahippocampal gyrus; ROC, receiver operating characteristic; SHAP, Shapley additive explanations; SVM, support vector machine; Thalamus_R, right thalamus; VAChT, vesicular acetylcholine transporter.

No significant spatial correlations were observed between regional AI values and neurotransmitter receptor or transporter density maps. In contrast, CFH abnormalities exhibited robust spatial associations with several key neurotransmitter systems (Figure 5A–C). Specifically, regional CFH alterations were significantly associated with the densities of the following targets: a negative correlation with 5‐hydroxytryptamine 2A receptors (5‐HT_2A_R; *r* = −0.4290, *p* < 0.001, Bonferroni corrected), and positive correlations with 5‐hydroxytryptamine 4 receptors (5‐HT_4_R; *r* = 0.4900), serotonin transporter (5‐HTT; *r* = 0.6438), α4β2 nicotinic acetylcholine receptors (A4B2; *r* = 0.4769), dopamine D_1_ receptors (D_1_R; *r* = 0.5199), dopamine D_2_ receptors (D_2_R; *r* = 0.6136), dopamine transporter (DAT; *r* = 0.5600), 6‐[^18^F]fluoro‐L‐DOPA uptake (F‐DOPA; *r* = 0.4936), N‐methyl‐D‐aspartate receptors (NMDAR; *r* = 0.6223), and vesicular acetylcholine transporter (VAChT; *r* = 0.6388). All associations survived Bonferroni correction for multiple comparisons (*p* < 0.001). These findings suggest that interhemispheric functional coordination, as indexed by CFH, may be modulated by the regional architecture of multiple neurotransmitter systems—particularly serotonergic, dopaminergic, glutamatergic, cholinergic, and monoaminergic pathways.

### 3.8 SVM‐Based Classification and SHAP‐Based Interpretation of AI/CFH Abnormalities

To further evaluate the discriminative capacity of regional AI and CFH features in differentiating RRD patients from HCs, an SVM classification model was employed. The model was optimized using a grid search strategy with an RBF kernel to determine the optimal hyperparameters. Model performance was assessed using LOOCV, with classification metrics including accuracy, sensitivity, specificity, precision, and the AUC. Among all input features, the SVM classifier based on CFH in the Thalamus_R achieved the highest classification performance, with an AUC of 0.8471 and an accuracy of 0.8046, indicating strong discriminative power (Figure 5D and Table 4). To enhance model interpretability, SHAP analysis was applied to quantify the contribution of each feature to the SVM decision function. SHAP value analysis revealed that both AI and CFH features contributed differentially to the classification task. As illustrated in the SHAP summary plots (Figure 5E–G), CFH in the right thalamus emerged as the most influential predictor, followed by CFH in the right occipital gyrus, CFH in the right parahippocampal gyrus, and AI in the right inferior frontal gyrus (opercular part). These findings highlight the pivotal role of CFH alterations in Thalamus_R in distinguishing RRD patients from HCs.

**TABLE 4:** SVM Classification Results for AI and CFH Abnormalities.

		AUC	Accuracy	Sensitivity	Specificity	Precious
AI	Frontal_Inf_Oper_R	0.8032	0.7241	0.5476	0.8889	0.8214
CFH	ParaHippocampal_R	0.7878	0.7241	0.6190	0.8222	0.7647
	Occipital_Inf_R	0.7746	0.7701	0.8333	0.7111	0.7292
	Thalamus_R	0.8471	0.8046	0.8333	0.7778	0.7778

Abbreviations: AI, autonomy index; AUC, area under the curve; CFH, connectivity between functionally homotopic voxels; Frontal_Inf_Oper_R, right inferior frontal gyrus, opercular part; HCs, healthy controls; Occipital_Inf_R, right inferior occipital gyrus; ParaHippocampal_R, right parahippocampal gyrus; RRD, rhegmatogenous retinal detachment; Thalamus_R, right thalamus.

### 3.9 Correlation Analysis Results

Spearman correlation analyses showed that neither AI nor CFH values were significantly associated with disease duration in patients with RRD. Specifically, the correlation between AI in Frontal_Inf_Oper_R and disease duration was *r* = 0.010 (*p* = 0.536), while the correlations between CFH values in ParaHippocampal_R, Occipital_Inf_R, and Thalamus_R with disease duration were *r* = 0.134 (*p* = 0.405), *r* = 0.221 (*p* = 0.164), and *r* = −0.074 (*p* = 0.645), respectively. In contrast, CFH in Thalamus_R showed a weak negative correlation with visual function (*r* = −0.326, *p* = 0.035), whereas no significant correlations were found between AI in Frontal_Inf_Oper_R (*r* = 0.135, *p* = 0.394), CFH in ParaHippocampal_R (*r* = −0.039, *p* = 0.805), or CFH in Occipital_Inf_R (*r* = −0.083, *p* = 0.603) and visual function. Detailed scatter plots illustrating these relationships are presented in Figures S3 and S4.

## 4 Discussion

This study is the first to systematically identify disruptions in hemispheric specialization and interhemispheric coordination in RRD patients using voxel‐level functional metrics: the AI and the CFH. Results revealed significantly increased CFH in the Thalamus_R, Occipital_Inf_R, and ParaHippocampal_R, along with elevated AI in the Frontal_Inf_Oper_R—indicating functional reorganization at both regional and interhemispheric levels. Spatial correspondence analysis using the AHBA showed that changes in AI and CFH closely matched regional gene expression profiles. These functional alterations were also significantly correlated with the spatial distribution of neurotransmitter receptors and transporters, particularly within serotonergic, dopaminergic, glutamatergic, and cholinergic systems. An SVM classification model, combined with SHAP‐based interpretability analysis, demonstrated the potential discriminative utility of AI and CFH, with CFH in the Thalamus_R emerging as the most critical feature for distinguishing RRD patients from HCs. In summary, this study proposes a novel multilevel framework integrating large‐scale functional reorganization, regional gene expression, and neurochemical architecture, providing new insight into the systemic effects of retinal detachment on the brain.

The significant changes in AI and CFH observed in RRD patients suggest that retinal detachment induces widespread reorganization involving both interhemispheric coordination and regional functional specialization. The increased CFH in the right thalamus is particularly notable. As a central hub in the visual pathway, the thalamus integrates retinal input and supports interhemispheric communication and attentional control [45, 46, 47]. This heightened connectivity may represent a compensatory response or disinhibition following loss of peripheral input, as the brain engages interhemispheric circuits to preserve functional balance. Similarly, elevated CFH in the right inferior occipital gyrus and parahippocampal gyrus is significant. The inferior occipital gyrus is involved in visual feature integration [48, 49], while the parahippocampal gyrus supports spatial scene recognition and complex perceptual tasks [50, 51, 52]. The enhanced connectivity in these areas may reflect the brain's attempt to reinforce bilateral coordination in response to unilateral visual disruption, maintaining visual integration. In contrast, the elevated AI in the opercular portion of the right inferior frontal gyrus suggests greater regional functional specialization. This region is associated with visual attention, executive control, and visuospatial processing [53, 54]; its increased functional autonomy may indicate adaptive reconfiguration of attentional networks following peripheral visual loss. Importantly, these results are consistent with previous neuroimaging findings in RRD and other ocular conditions. Earlier studies have documented reorganization in the occipital, parietal, and primary visual cortices in RRD patients, indicating that the brain rapidly adapts to disrupted sensory input [8, 55, 56]. Altered thalamic connectivity has also been reported in glaucoma and retinitis pigmentosa, reinforcing the thalamus's role as a key mediator of visual cortical plasticity [57, 58]. However, prior research has largely focused on static or localized connectivity and structural changes, with little emphasis on core organizational principles like specialization and interhemispheric integration. From a broader functional perspective, understanding whether these alterations reflect adaptive reorganization or maladaptive hypersynchrony is crucial for elucidating the plasticity mechanisms following retinal detachment. Notably, in our study, the changes in AI and CFH were not systematically correlated with disease duration or visual function, except for a weak negative correlation between right thalamic CFH and visual acuity. This pattern does not exhibit a linear dependence on clinical severity but rather suggests a recalibration mechanism, whereby the thalamus enhances interhemispheric coordination to maintain functional balance following peripheral input loss. As a central hub in the visual pathway, the thalamus may exert dynamic regulatory effects to preserve large‐scale network stability under conditions of sensory disruption. The absence of strong correlations in other regions further indicates that the observed network reorganization is not solely driven by peripheral pathology or disease progression, but may instead represent higher‐order adaptive modulation aimed at maintaining visual–cognitive integration. Collectively, these findings support a model of dynamic plasticity characterized by “bottom‐up disruption and top‐down regulation,” underscoring the thalamus's pivotal role in interhemispheric functional recalibration.

To further elucidate the potential molecular underpinnings of altered hemispheric specialization and interhemispheric cooperation in RRD patients, we spatially aligned the brain maps of AI and CFH with the AHBA transcriptomic database. PLS regression revealed that the PLS1 accounted for 52.4% and 59.2% of the spatial variance in AI and CFH differences, respectively, suggesting a strong coupling between RRD‐related functional reorganization and regional gene expression gradients. Subsequent analysis identified a subset of genes with significantly positive or negative loadings in PLS1, potentially representing the regional molecular substrates driving the observed AI and CFH alterations. Functional annotation of these genes revealed marked pathway specificity: genes associated with AI were enriched in processes such as trans‐synaptic signaling, presynaptic structure regulation, sensory organ development, and response to exogenous stimuli. These findings are consistent with previous studies implicating genes such as OLFM1 and SYP in synaptic maintenance, neural transmission, and sensory processing [59, 60, 61, 62]. Conversely, genes associated with CFH were enriched in pathways involving Notch signaling, postsynaptic structure, and axonal transport—mechanisms widely implicated in cortical network stability and metabolic support [63, 64, 65, 66]. PPI network analysis further revealed several biologically meaningful gene modules. AI‐associated modules were enriched in synaptic vesicle cycling and chromatin remodeling pathways, suggesting that functional specialization may depend on both synaptic efficiency and epigenetic regulation. In contrast, CFH‐associated modules were primarily involved in mitochondrial metabolism, autophagy, transcriptional regulation, and cytoskeletal dynamics—highlighting the energy and structural demands underlying interhemispheric integration. Temporal‐specific expression patterns of representative genes provided additional support for their functional relevance. OLFM1 has been shown to play a critical role in synaptogenesis and circuit stabilization [67, 68], whereas CASP10 may contribute to neural circuit refinement during development by mediating programmed cell death [69, 70]. Both genes exhibited peak expression in the neocortex and hippocampus from adolescence to early adulthood, suggesting their involvement in synaptic remodeling and large‐scale network reconfiguration during key developmental windows. In contrast, FAM114A1 and R3HDM1, implicated in signaling regulation and RNA metabolism, respectively [71, 72, 73], displayed stable expression across brain regions, pointing to their potential roles in maintaining long‐term neural homeostasis. Taken together, transcriptomic and PPI network analyses converge on a molecular framework encompassing synaptic regulation, developmental plasticity, and homeostatic maintenance. These findings provide mechanistic insight into the region‐ and function‐specific vulnerability reflected by AI and CFH alterations and suggest that RRD‐related functional reorganization may be driven by the coordinated activity of genes with spatial and temporal expression specificity.

This study further examined the neurochemical basis of functional abnormalities by integrating spatial maps of neurotransmitter receptors and transporters. While AI did not show significant associations with neurotransmitter density, CFH variations were consistently linked to several major neurotransmitter systems. These results suggest that interhemispheric coordination is inf luenced not only by structural connectivity and network topology [74], but also by neurochemical modulation [15]. Specifically, CFH was negatively correlated with 5‐HT_2_A receptor density and positively correlated with 5‐HT_4_ receptors and 5‐HTT. This pattern may reflect serotonin's bidirectional role in regulating interregional communication, cortical synchrony, and cognitive flexibility [75, 76]. CFH also showed positive associations with dopaminergic markers, including D_1_ and D_2_ receptors, F‐DOPA uptake, and DAT density. D_1_ receptors have been linked to hippocampal plasticity [77], while both D_1_ and D_2_ receptors are involved in motor inhibition and attentional control [78]. F‐DOPA, a marker of dopamine synthesis—especially in the striatum [79]—further implicates the dopaminergic system in attention and interhemispheric plasticity. CFH abnormalities were also positively correlated with glutamatergic and cholinergic systems, particularly through NMDA receptors, A4B2 receptors, and VAChT. NMDA receptors, central to excitatory glutamatergic signaling, play critical roles in synaptic plasticity, information integration, and coordination of large‐scale brain networks [80]. The cholinergic system contributes to attention regulation, signal amplification, and cortical excitability via A4B2 receptors and VAChT [81, 82, 83]. These findings suggest that glutamatergic and cholinergic systems may work together to regulate interhemispheric integration and functional coordination. Although spatial correlations do not establish causality, they provide a valuable foundation for understanding the neurochemical basis of altered interhemispheric connectivity and identifying potential molecular targets for future interventions.

To assess the ability of AI and CFH to distinguish individual RRD patients from healthy controls, we employed an SVM classifier. The model based on CFH in the right thalamus achieved the highest performance, with an AUC of 0.8471 and an accuracy of 0.8046, indicating robust classification ability. These results reinforce the central role of the Thalamus_R in interhemispheric coordination and highlight its potential as an imaging indicator of brain alterations linked to retinal disease. To address the “black‐box” nature of SVMs, we used SHAP to determine the contribution of each feature to the model's predictions. SHAP analysis identified CFH in the Thalamus_R as the most influential predictor, followed by CFH in the Occipital_Inf_R, ParaHippocampal_R, and AI in the Frontal_Inf_Oper_R. These findings align with previously observed functional abnormalities and emphasize the importance of hub regions involved in interhemispheric connectivity. Notably, combining SVM with SHAP has become a valuable strategy in neuroimaging, aiding early detection, subtype classification, and personalized prediction in disorders such as major depressive disorder [84, 85], mild traumatic brain injury [86], and Parkinson's disease [87]. This is the first study to incorporate AI and CFH into an SVM‐SHAP framework for classifying an ophthalmic condition, offering a novel approach for quantitatively identifying brain dysfunction associated with retinal pathology.

The present findings have potential translational implications for early detection and rehabilitation of RRD. The observed alterations in hemispheric specialization and interhemispheric coordination—particularly the increased CFH in the thalamus and occipital regions—may reflect compensatory reorganization that occurs before irreversible visual or cortical degeneration. Thus, CFH‐based neuroimaging markers could serve as sensitive indicators for detecting early‐stage neural dysfunction associated with retinal detachment, even prior to overt clinical symptoms. Furthermore, these findings may inform the development of targeted neuromodulatory or rehabilitation strategies aimed at enhancing interhemispheric communication and restoring visual network balance following retinal reattachment surgery. For instance, non‐invasive brain stimulation or neurofeedback training directed toward thalamic and occipital circuits may facilitate neural plasticity and improve visual recovery outcomes. Together, this study bridges fundamental brain–retina mechanisms with clinical translation, highlighting new possibilities for early intervention and individualized rehabilitation in patients with RRD.

## 5 Limitations

This study is the first to apply voxel‐level AI and CFH metrics in RRD patients, systematically uncovering patterns of reorganization in hemispheric specialization and interhemispheric coordination. By integrating multimodal molecular data, we developed a unified framework that links brain function with gene expression and neurotransmitter systems. However, several limitations should be acknowledged. First, due to the low incidence of RRD, the relatively small sample size—although consistent with many previous neuroimaging studies—may constrain external validity and introduce uncertainty into statistical inferences. A post hoc power analysis assuming a medium effect size (Cohen's *d* = 0.5) yielded a power of 0.6346, which is below the conventional threshold of 0.80, underscoring the need for future studies with larger and more adequately powered samples. Second, the cross‐sectional design restricts causal inference, limiting conclusions about the temporal dynamics of RRD‐induced brain changes. Third, clinical heterogeneity related to the number of retinal tears and the extent of retinal detachment was not further stratified in the present analysis. Although all patients met the diagnostic criteria for RRD, variations in retinal break characteristics might have introduced additional variance in the observed neuroimaging results. Future studies with larger and more stratified samples should examine whether these clinical factors correspond to distinct AI and CFH alteration patterns. Fourth, transcriptomic and receptor density data were obtained from healthy cohorts (AHBA and PET databases), which, while informative, may not accurately capture disease‐specific molecular alterations. Fifth, due to limited right‐hemisphere sampling in the AHBA, gene expression analyses were confined to the left hemisphere, possibly missing bilateral effects of RRD. Sixth, although the SVM + SHAP framework demonstrated good interpretability and classification performance, the relatively small sample size may still pose a potential risk of overfitting. To mitigate this issue, we employed several methodological safeguards—including grid search optimization, selection of the RBF kernel, and LOOCV—to ensure model robustness and minimize information leakage. Nevertheless, the absence of an external validation cohort limits the generalizability of the present findings. Future studies should incorporate independent datasets, permutation testing, and bootstrap resampling to further verify model stability, enhance reproducibility, and confirm the reliability of the identified discriminative features. Finally, future studies should aim to expand sample sizes, adopt longitudinal designs, and implement multicenter, multimodal research strategies to strengthen the robustness and generalizability of findings. Moreover, integrating patient‐specific transcriptomic profiles with diverse molecular imaging modalities may help elucidate the pathophysiological mechanisms and interindividual variability in RRD, thereby advancing precision diagnosis and targeted interventions.

## 6 Conclusion

In summary, this study systematically identified aberrant hemispheric specialization and interhemispheric coordination in patients with RRD using voxel‐level AI and CFH metrics. By integrating transcriptomic and neurochemical data, we revealed that these abnormalities are associated with gene expression profiles and neurotransmitter systems involved in synaptic signaling, Notch regulation, and neuroplastic remodeling. Furthermore, the SVM–SHAP model demonstrated the potential discriminative utility of these features, with the right thalamus emerging as a key hub of functional reorganization. Collectively, these findings provide novel insight into the neurobiological mechanisms underlying RRD‐induced brain functional remodeling and offer theoretical support and a methodological foundation for developing central intervention strategies and potential discriminative imaging tools for retinal diseases.

## Author Contributions

Yu Ji conceived the study, performed the statistical analyses, and drafted the initial manuscript. Yuan‐Yuan Wang undertook comprehensive manuscript editing, including critical language refinement and structural review. Yu Ji also led data acquisition and conducted subsequent validation to ensure dataset completeness. Xiao‐Rong Wu contributed extensively to protocol development, led the neuroimaging analyses based on MRI data, and provided clinical oversight. In addition, Xiao‐Rong Wu offered strategic guidance on study design and coordinated interdisciplinary collaboration across research domains. All authors reviewed and revised the manuscript for important intellectual content, approved the final version for publication, and take full responsibility for the integrity of the work.

## Funding

We gratefully acknowledge the support of the National Natural Science Foundation of China (82160207), the Key Projects of Jiangxi Youth Science Fund (20202ACBL216008), and the Science and Technology Plan of the Jiangxi Provincial Health and Health Commission (202130156).

## Ethics Statement

The trial was approved by the ethics committee of the First Affiliated Hospital of Nanchang University. All methods are performed in accordance with relevant guidelines and regulations.

## Consent

Informed consent from participants to participate has been obtained.

## Conflicts of Interest

The authors declare no conflicts of interest.

## Supporting information


**Figure S1:** PPI network analysis of genes associated with AI and CFH alterations.
**Figure S2:** Spatiotemporal expression trajectories of representative genes associated with AI and CFH alterations.
**Figure S3:** Scatter plots showing the Spearman correlations between vision and imaging‐derived metrics in brain regions that exhibited significant group differences.
**Figure S4:** Scatter plots showing the Spearman correlations between disease duration and imaging‐derived metrics in brain regions that exhibited significant group differences.

**Table S1:** Information about the six donors in AHBA.

**Table S2:**(A) Genes and PLS1+ weights from the PLS correlation between AI t‐map and gene expression. (B) Genes and PLS1− weights from the PLS correlation between AI t‐map and gene expression. (C) Genes and PLS1+ weights from the PLS correlation between CFH t‐map and gene expression. (D) Genes and PLS1− weights from the PLS correlation between CFH t‐map and gene expression.

## Data Availability

The data that support the findings of this study are available from the corresponding author upon reasonable request.
