# Supplementary material for: Altered Functional Specialization and Interhemispheric Coordination in Rhegmatogenous Retinal Detachment: Associations With Gene Expression, Neurotransmitter Receptor Distribution, and SVM–SHAP Classification: A Multimodal Neuroimaging–Transcriptomics Study Integrating Functional Metrics and Interpretable Machine Learning
Source: CNS Neurosci Ther. 2026 Jan 7;32(1):e70678. doi: 10.1002/cns.70678 (PMC12775831; doi:10.1002/cns.70678)
Supplement: Supplementary file 1 — Figure S1: PPI network analysis of genes associated with AI and CFH alterations. Figure S2: Spatiotemporal expression trajectories of representative genes associated with AI and CFH alterations. Figure S3: Scatter plots showing the Spearman correlations between vision and imaging‐derived metrics in brain regions that exhibited significant group differences. Figure S4: Scatter plots showing the Spearman correlations between disease duration and imaging‐derived metrics in brain regions that exhibited significant group differences. [file CNS-32-e70678-s002.docx]

(1) PPI Network Analysis of Genes Associated with Changes in AI and CFH:


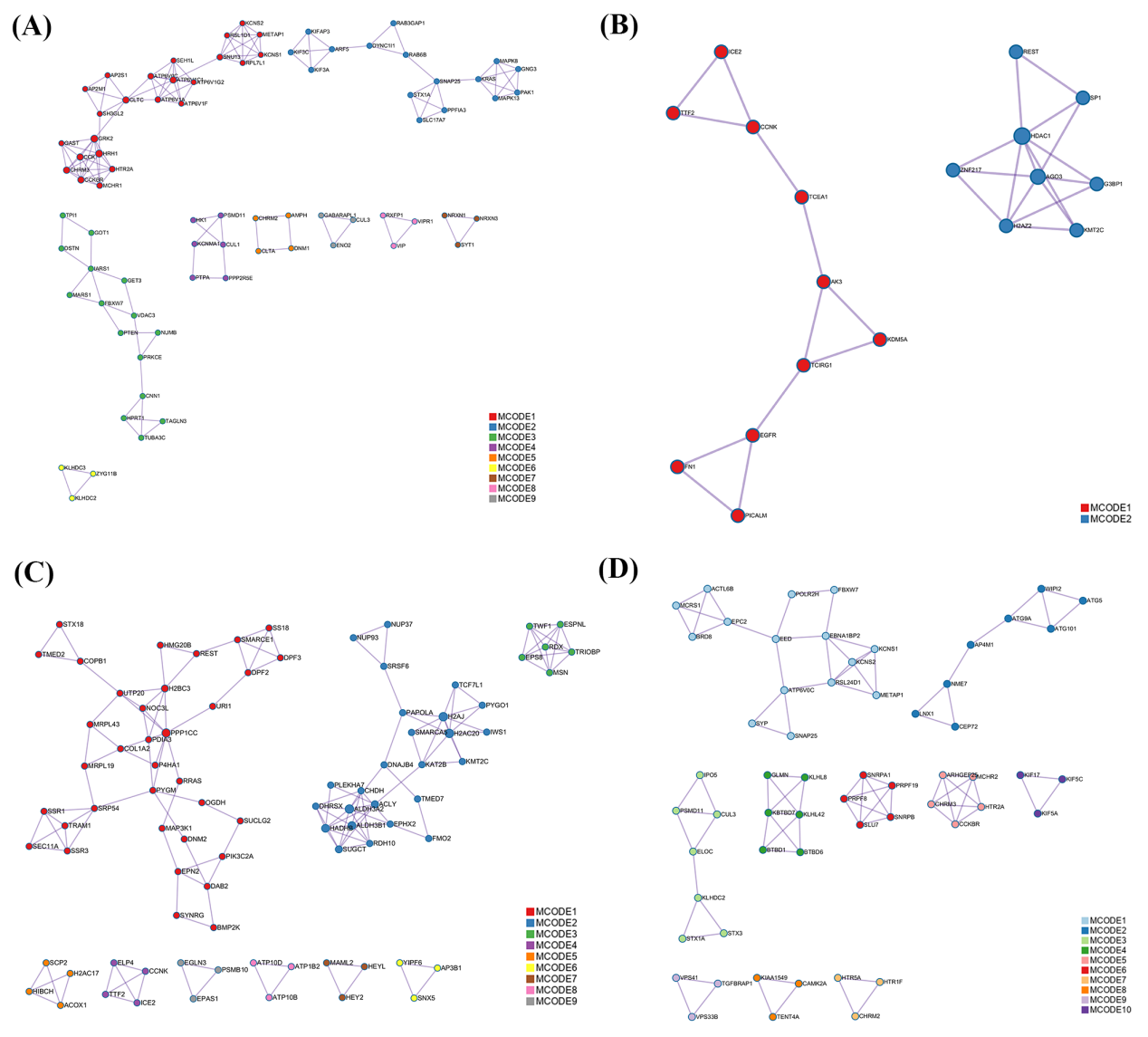


Supplementary Figure S1. PPI network analysis of genes associated with AI and CFH alterations.

(A–B) PPI networks of AI-associated genes.

1. Network constructed from the PLS1⁺ gene set, clustered into nine functional modules using MCODE.

(B) Network constructed from the PLS1⁻ gene set, revealing two distinct functional clusters.

(C–D) PPI networks of CFH-associated genes.

(C)Network derived from the PLS1⁺ gene set, comprising nine MCODE-identified functional clusters.

(D) Network derived from the PLS1⁻ gene set, consisting of multiple interconnected functional modules.

Each node represents a protein encoded by the corresponding gene, and edges indicate experimentally validated or predicted protein–protein interactions. Different colors correspond to distinct MCODE clusters identified by Metascape.

Abbreviations: AI, autonomy index; CFH, connectivity between functionally homotopic voxels; PLS, partial least squares; PLS1, first PLS component; MCODE, Molecular Complex Detection; PPI, protein–protein interaction.

(2)Temporal-specific expression analysis of representative genes:


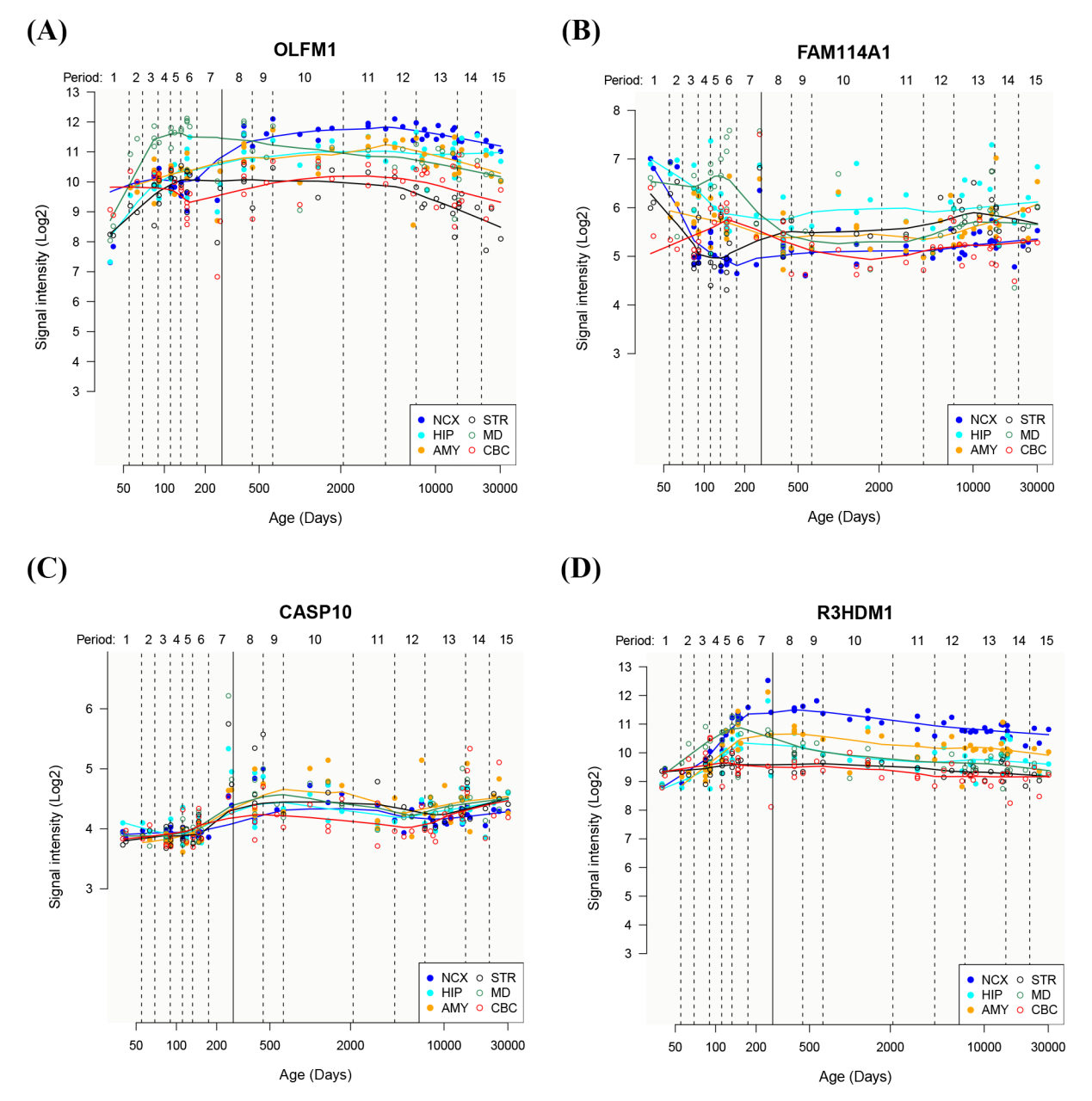


Supplementary Figure S2. Spatiotemporal expression trajectories of representative genes associated with AI and CFH alterations.

(A–B) Expression patterns of AI-associated genes.

(A) OLFM1 (AI–PLS1⁺) shows high expression in the neocortex (NCX) and hippocampus (HIP), increasing after birth and stabilizing during adolescence.

(B) FAM114A1 (AI–PLS1⁻) exhibits moderate expression early in development followed by stabilization across regions.

(C–D) Expression patterns of CFH-associated genes.

(C) CASP10 (CFH–PLS1⁺) displays low but widespread expression with a slight increase during postnatal development.

(D) R3HDM1 (CFH–PLS1⁻) shows early upregulation and sustained high expression, particularly in the neocortex (NCX).

Each plot covers 15 developmental periods across six brain regions (NCX, HIP, AMY, STR, MD, CBC). Y-axis: log₂ signal intensity; X-axis: age (days).

Abbreviations: AI, autonomy index; CFH, connectivity between functionally homotopic voxels; PLS, partial least squares; PLS1, first PLS component.

(3)Spearman correlation analysis between vision and brain imaging metrics (AI and CFH):


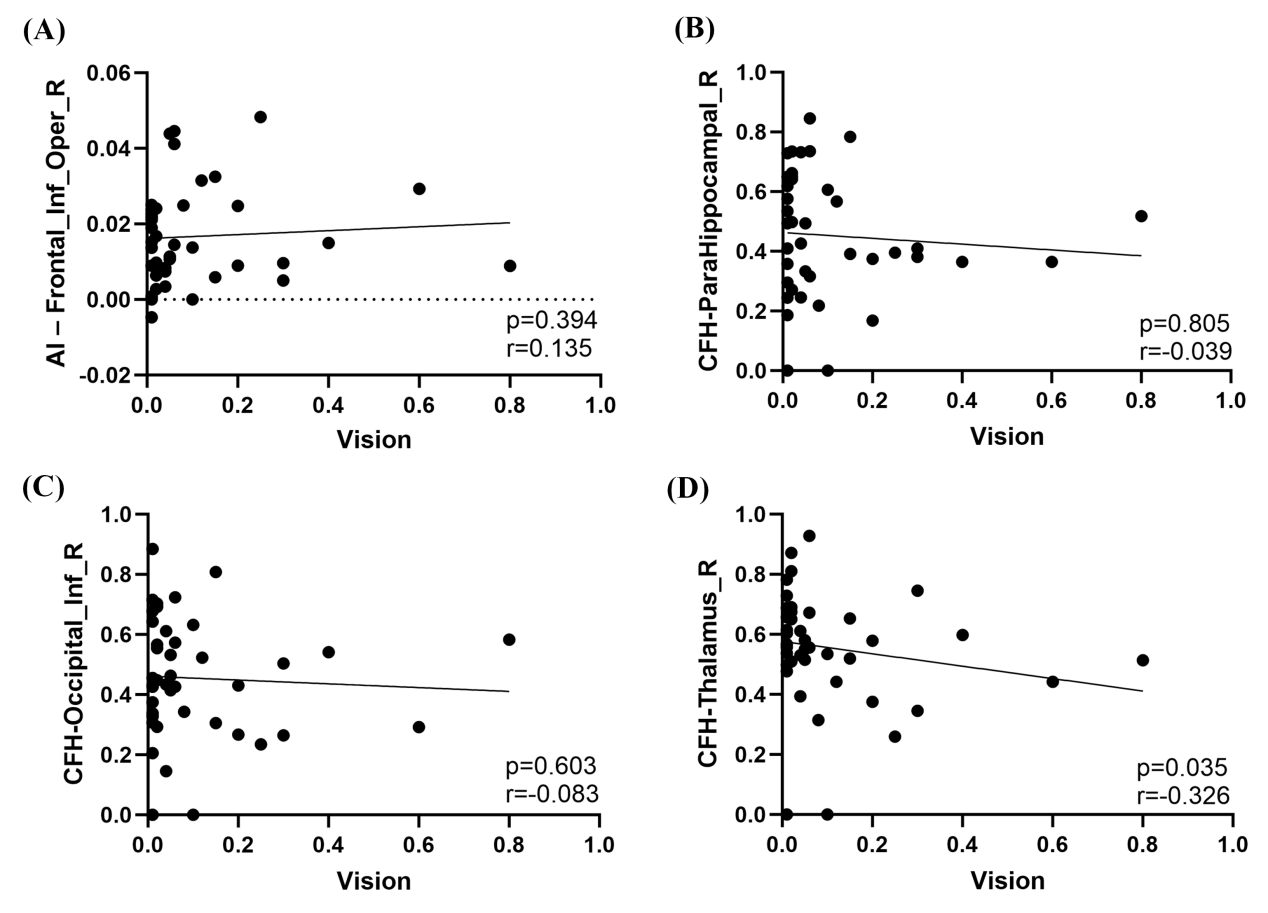


Supplementary Figure S3. Scatter plots showing the Spearman correlations between vision and imaging-derived metrics in brain regions that exhibited significant group differences.

(A) Correlation between AI in the right inferior frontal operculum (AI–Frontal_Inf_Oper_R) and vision.

(B) Correlation between CFH in the right parahippocampal gyrus (CFH–ParaHippocampal_R) and vision.

(C) Correlation between CFH in the right inferior occipital gyrus (CFH–Occipital_Inf_R) and vision.

(D) Correlation between CFH in the right thalamus (CFH–Thalamus_R) and vision.

Each dot represents one RRD patient. Correlation coefficients (r) and p-values were calculated using Spearman’s rank correlation test. A significant negative correlation was observed between CFH in the right thalamus and vision (p = 0.035, r = −0.326), whereas no significant correlations were found in other regions.

(4)Spearman correlation analysis between disease duration and brain imaging metrics (AI and CFH):


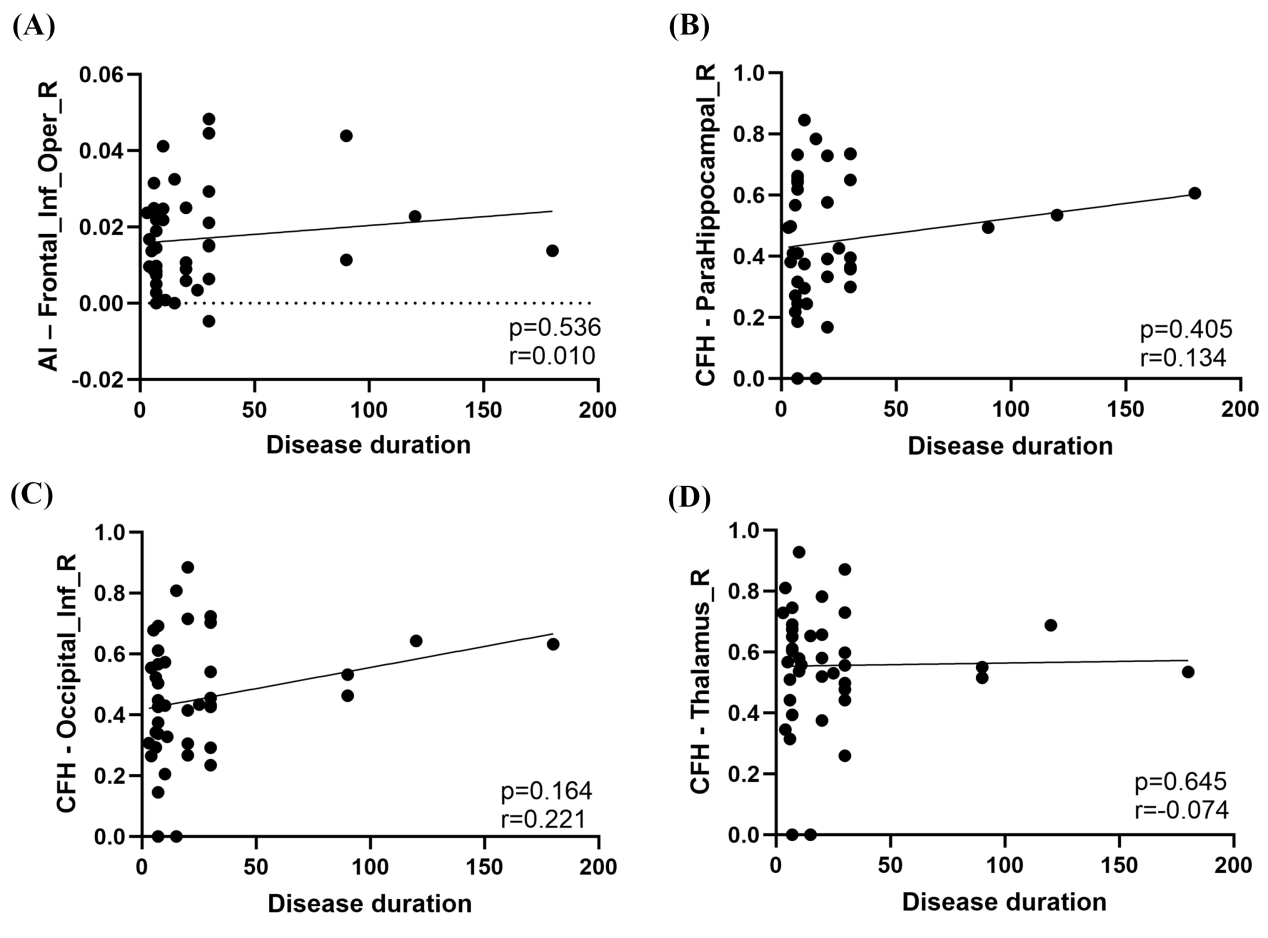


Supplementary Figure S4. Scatter plots showing the Spearman correlations between disease duration and imaging-derived metrics in brain regions that exhibited significant group differences.

(A) Correlation between AI in the right inferior frontal operculum (AI–Frontal_Inf_Oper_R) and disease duration.

(B) Correlation between CFH in the right parahippocampal gyrus (CFH–ParaHippocampal_R) and disease duration.

(C) Correlation between CFH in the right inferior occipital gyrus (CFH–Occipital_Inf_R) and disease duration.

(D) Correlation between CFH in the right thalamus (CFH–Thalamus_R) and disease duration.

Each dot represents one RRD patient. Correlation coefficients (r) and p-values were computed using Spearman’s rank correlation test. No significant correlations were found between disease duration and any of the imaging metrics.
