# Supplementary material for: Altered Functional Specialization and Interhemispheric Coordination in Rhegmatogenous Retinal Detachment: Associations With Gene Expression, Neurotransmitter Receptor Distribution, and SVM–SHAP Classification: A Multimodal Neuroimaging–Transcriptomics Study Integrating Functional Metrics and Interpretable Machine Learning
Source: CNS Neurosci Ther. 2026 Jan 7;32(1):e70678. doi: 10.1002/cns.70678 (PMC12775831; doi:10.1002/cns.70678)
Supplement: Supplementary file 2 — Table S1: Information about the six donors in AHBA. [file CNS-32-e70678-s001.docx]

| **Table S1. Information about the six donors in AHBA.** | | | | | |
| --- | --- | --- | --- | --- | --- |
| **Donor** | **Age** | **Sex** | **Ethnicity** | **Hemisphere** | **Post-mortem interval ^a^** |
| H0351 1009 | 57 | Male | Caucasian | L | 25.5 h |
| H0351 1012 | 31 | Male | Caucasian | L | 17.5 h |
| H0351 1015 | 49 | Female | Hispanic | L | 30 h |
| H0351 1016 | 55 | Male | Caucasian | L | 18 h |
| H0351 2001 | 24 | Male | African American | L + R | 23 h |
| H0351 2002 | 39 | Male | African American | L + R | 10 h |
| a Post-mortem interval is defined as the time period from the time of death to the time the tissue is frozen.  Abbreviations: AHBA, Allen Human Brain Atlas; L, left; R, Right. | | | | | |
